# EHMTI-0251. Brief intervention for medication-overuse headache in primary care - 1-year follow-up – the BIMOH study

**DOI:** 10.1186/1129-2377-15-S1-C31

**Published:** 2014-09-18

**Authors:** ES Kristoffersen, J Straand, KG Vetvik, MB Russell, C Lundqvist

**Affiliations:** 1 Department of General Practice, University of Oslo, Oslo, Norway; 2 Head and Neck Research Group Research Centre, Akershus University Hospital, Lørenskog, Norway; 3 Research Centre, Akershus University Hospital, Lørenskog, Norway

## Introduction

MOH can be identified in the general population through simple screening for headache frequency followed by the Severity of Dependence Scale (SDS).

## Aim

To evaluate the long-term effectiveness of brief intervention (BI) for medication-overuse headache (MOH) in primary care.

## Methods

This was a double-blind pragmatic cluster randomised parallel controlled trial in primary care in Norway. Fifty GPs were randomised to receive BI training or to continue their business as usual (BAU). 25 486 patients aged 18-50 years from the GPs lists were screened for MOH by a questionnaire. Patients were cluster randomised and received treatment by their GP. GPs practising BI assessed their MOH patients using the SDS. Based on this, the patients received feedback about the risk of MOH, and recommendations for reducing intake of headache medication. Outcomes were interview based and assessed one year after inclusion in the study.

## Results

Sixty MOH patients were included at baseline and 57 patients followed-up after one year. Analyses of the outcomes showed that BI was better than BAU with significant improvements only in the BI group at three months which persisted up to one year. Only two initially detoxified patients relapsed into medication overuse after one year. More results are currently being analysed and will be presented at the meeting.

## Conclusion

BI intervention for MOH conducted in primary care has significant effects lasting over twelve months.

No conflict of interest.

